# Broadband photon-counting dual-comb spectroscopy with attowatt sensitivity over turbulent optical paths

**DOI:** 10.1038/s41377-025-01934-7

**Published:** 2025-08-27

**Authors:** Wei Zhong, Yingyu Liu, Qin Yin, Ruocan Zhao, Chong Wang, Wei Ren, Xiankang Dou, Xianghui Xue

**Affiliations:** 1 CAS Key Laboratory of Geospace Environment, School of Earth and Space Sciences, University of Science and Technology of China, 230026 Hefei, China; 2 Department of Mechanical and Automation Engineering, The Chinese University of Hong Kong, New Territories, Hong Kong SAR China; 3 Hefei National Laboratory, University of Science and Technology of China, 230088 Hefei, China; 4 Hefei National Research Center for Physical Sciences at the Microscale and School of Physical Sciences, University of Science and Technology of China, 230026 Hefei, China; 5 CAS Center for Excellence in Comparative Planetology, Anhui Mengcheng Geophysics National Observation and Research Station, University of Science and Technology of China, 230026 Hefei, China; 6 Present Address: Department of Mechanical and Automation Engineering, The Chinese University of Hong Kong, New Territories, Hong Kong SAR China

**Keywords:** Near-infrared spectroscopy, Optical sensors

## Abstract

Photon-counting dual-comb spectroscopy (DCS) opens new possibilities for deploying DCS in scenarios previously constrained by limited detection sensitivity. However, inevitable optical path fluctuations hinder its practical implementation. Here, we propose a method to ensure the long-term stability of photon-counting DCS, overcoming turbulent optical paths, achieving attowatt-level detection sensitivity and quick acquisition times. Using a compact all-fiber dual-comb system, we achieve 20 nm broadband DCS of H^13^C^14^N across the C-band with an average detected power of only 4 attowatts per-comb line. Despite significant vibrations throughout measurements, the spectra maintain comb-line resolution and shot-noise-limited signal-to-noise ratios. Additionally, the system demonstrates successful deployment in open-path measurements, overcoming 93 dB attenuation. Our approach enables remote sensing of CO_2_, H_2_O, and HDO over a continuous 20-h observation period. This method highlights the potential for applications in fields such as metrology, quantum physics, and atmospheric sensing, especially in turbulent environments like open air or water, within a field-deployable system.

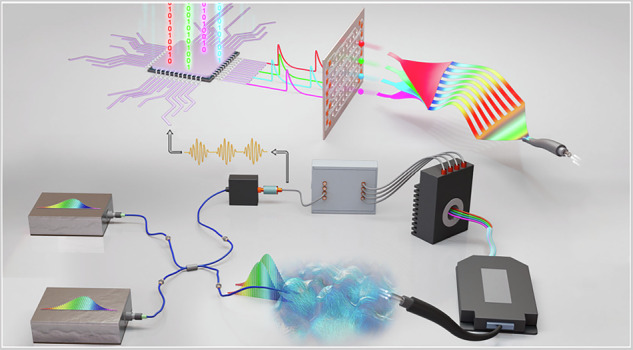

## Abstract (web summary)

An all-fiber photon-counting system enables robust, field-deployable dual-comb spectroscopy under challenging conditions.

## Introduction

Dual-comb spectroscopy (DCS) has emerged as a transformative tool in precision spectroscopy, using two optical frequency combs (OFCs) with a slight repetition frequency difference to enable high-precision and high-resolution measurements across a broad spectral range without any mechanical scanning components^1–4^. However, DCS faces limitations in detection sensitivity, particularly under conditions of inherently low detection power^5–10^, or when trying to access high power and coherence across specific spectral ranges^11–16^. Photon-counting dual-comb interferometry is promising to address these challenges^17^, which harnesses photon clicks captured by a single-photon detector (SPD) for statistical reconstruction of broadband spectral information. This method has been recently extended to applications such as ranging and imaging^18,19^. Linear gas-phase absorption spectroscopy, which demands high resolution to resolve narrow absorption features with optimal signal-to-noise ratio (SNR)^20^, has also been demonstrated^21^. However, these studies often involve a trade-off between resolution, precision, SNR, and spectral bandwidth. Meanwhile, these methods have been constrained to spectral regions suitable for detectors that support high count rates and low dark counts, i.e., photomultiplier tubes (PMTs) for the near-ultraviolet, Si single-photon avalanche photodiodes (Si-SAPDs) for the visible, or bulky and expensive superconducting nanowire single-photon detectors (SNSPDs) for the near-infrared, enabling the reconstruction of the desired information over relatively short durations. In specific DCS research areas such as the near-infrared, InGaAs-based SPADs are appealing due to their practicality, portability and cost-effectiveness. Realizing open-path photon-counting DCS by these compact and low-cost SPADs could unlock new applications, such as open-path DCS^22–26^ over ultra-long open-path or non-cooperative configuration without the retro-mirror within a portable and low-power consumption system. However, InGaAs-SPADs face limitations in the maximal count rate and often exhibit higher dark counts^27–30^. Hence, achieving high-SNR broadband spectra necessitates prolonged accumulation times, making long-term stability a crucial factor for optimal spectral results.

Traditional DCS requires a high mutual coherence between dual-comb sources to support high SNR and comb-mode-resolved spectrum through coherent averaging^31–34^. Nevertheless, this falls short in photon-counting DCS. Photon-counting DCS relies on first-order temporal detection of single-photon interference among multi-mode coherent states across different frequencies generated from two independent comb sources. The first-order interference signal is particularly sensitive to path length fluctuations, making it difficult to observe^35–37^, especially when extended observation times are required for dual-comb interference involving thousands of frequency lines. Optical phase noise in practical scenarios, such as those induced by fiber movements, open-air turbulence, and flowing liquids, which are inevitable, random, and uncontrollable, significantly challenge long-term stability, thereby reducing the effective extraction of dual-comb single-photon interference from photon events.

To address these challenges, we propose a common-mode sensing configuration and a start-signal-triggered photon-counting protocol, which ensures robust, long-term photon-counting stability for practical remote sensing applications. Despite considerable optical path fluctuations, our proposed configuration, as shown in experiments and analyses in subsequent sections, achieves attowatt-level DCS with comb-mode resolution and shot-noise-limited SNR. This stable photon-counting approach allows for photon-counting DCS under the widest bandwidths and lowest detection power levels yet recorded. This method is demonstrated for open-path photon-counting DCS for the first time. We conclude with a forward-looking perspective on the potential applications of this technique in metrology and remote sensing under extreme conditions.

## Results

### Principle

**Fig. 1:**
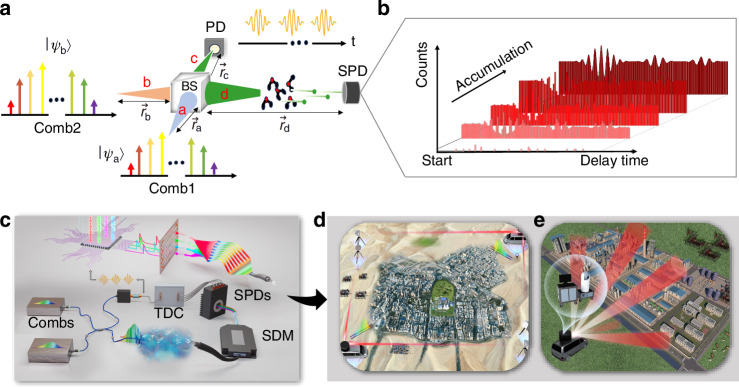
Principle of photon-counting dual-comb spectroscopy immune to optical path fluctuations. BS beam splitter, PD photodetector, SPD single-photon detector, TDC time-to-digital converter, SDM spectral division multiplexer. **a** Conceptual schematic of single-photon interference with two frequency combs. The two combs with a slight repetition frequency difference are combined via a beam splitter. Discrete photon clicks are captured by a single-photon detector under light-starving conditions. **b** Principle of photon-counting statistics. The timing statistics of detected photon clicks reveal single-photon dual-comb interference, which contains spectral information. **c** Schematic of the proposed all-fiber photon-counting DCS system, which is immune to optical path fluctuations. The workflow diagram shows the segmented parallel detection process. This field-deployable system is suitable for challenging applications, such as large-scale open-path environmental monitoring (**d**), and non-cooperative open-path DCS (**e**) without the need for retroreflectors

The principle of dual-comb single-photon interference with the proposed common-mode sensing configuration and photon-counting statistics is depicted in Fig. 1. As shown in Fig. 1a, the optical path can be divided into two categories determined by a beam splitter (BS): the internal system paths represented by ‘a’, ‘b’ and ‘c’ (with length *r*_a_, *r*_b_, *r*_c_), and the sensing path corresponding to the path ‘d’ (with length ${r}_{{\rm{d}}}$). The quantum states of the light fields emitted by the two OFCs, can be expressed as $\left|{\psi }_{{\rm{a}}}\right\rangle =\left|{\alpha }_{0}\right\rangle \left|{\alpha }_{1}\right\rangle \cdots \left|{\alpha }_{N-1}\right\rangle$ and $\left|{\psi }_{{\rm{b}}}\right\rangle =\left|{{\alpha }^{{\prime} }}_{0}\right\rangle \left|{{\alpha }^{{\prime} }}_{1}\right\rangle \cdots \left|{{\alpha }^{{\prime} }}_{N-1}\right\rangle$; $\left|{\alpha }_{i}\right\rangle$ and $\left|{\alpha }_{i}{\prime} \right\rangle$ (*i* = 0, 1, …, *N*–1) represent the coherent states of the frequency modes ${\omega }_{i}$ and ${\omega {\prime} }_{i}$, respectively; ${\omega }_{i}={\omega }_{{\rm{a}}}+i\cdot 2{\rm{\pi }}{f}_{{\rm{r}}}$ and ${\omega {\prime} }_{i}={\omega }_{{\rm{b}}}+i\cdot {2{\rm{\pi }}\cdot (f}_{{\rm{r}}}+\varDelta {f}_{{\rm{r}}})$. Here, ${\omega }_{{\rm{a}}}$ and ${\omega }_{{\rm{b}}}$ denote the lowest angle frequencies of the two OFCs, and ${f}_{{\rm{r}}}$ and ${f}_{{\rm{r}}}+\varDelta {f}_{{\rm{r}}}$ represent the repetition frequencies. The frequency difference between the coherent state pair $\left|{\alpha }_{i}\right\rangle$ and $\left|{\alpha }_{i}{\prime} \right\rangle$ is thus $\Delta {\omega }_{i}={\omega {\prime} }_{i}-{\omega }_{i}={\omega }_{0}+i\cdot 2{\rm{\pi }}{\varDelta f}_{{\rm{r}}}$, with ${\omega }_{0}={\omega }_{{\rm{b}}}-{\omega }_{{\rm{a}}}$. The detection probability at the output path ‘d’, denoted as ${P}_{{\rm{d}}}\left(t\right)$, is proportional to the expectation value of the field operator, which includes an interference term between all coherent state pairs $\left|{\alpha }_{i}\right\rangle$ and $\left|{\alpha }_{i}{\prime} \right\rangle$. Without considering dispersion, this interference term can be expressed as (see Supplementary Note 1):

1 $${\widetilde{V}}_{{\rm{d}}}\left(t\right)=\mathop{\sum }\limits_{i=0}^{N-1}{\alpha }_{i}^{* }\cdot {\alpha }_{i}^{{\prime}}\cdot{e}^{-\left({\chi }_{i}^{* }+{\chi }_{i}^{{\prime} }\right)}\cdot {e}^{j\cdot \left[\left({\omega }_{0}+i\cdot 2{\rm{\pi }}\varDelta {f}_{{\rm{r}}}\right)\left(t+\frac{{r}_{{\rm{d}}}+{r}_{{\rm{a}}}}{c}+\frac{{f}_{{\rm{r}}}+\varDelta {f}_{{\rm{r}}}}{\varDelta {f}_{{\rm{r}}}}\cdot \frac{\varDelta r}{c}\right)+\varOmega \right]}$$

Where $\varDelta r=|{r}_{{\rm{a}}}-{r}_{{\rm{b}}}|$ is the path length difference before the two combs combine; *c* is the group velocity of comb light; and $\varOmega =\frac{\varDelta r}{c}\cdot ({\omega }_{{\rm{b}}}-{\frac{{f}_{{\rm{r}}}+\varDelta {f}_{{\rm{r}}}}{\varDelta {f}_{{\rm{r}}}}\cdot \omega }_{0})$. The term ${\alpha }_{i}^{* }\cdot {\alpha }_{i}^{{\prime} }\cdot {e}^{-\left({\chi }_{i}^{* }+{\chi }_{i}^{{\prime} }\right)}$ accounts for the effect of interaction between materials and light along the path. Equation (1) indicates that single-photon interference could occur between two independent broadband coherent light sources, forming the fundamental prerequisite for DCS detection under light-starving conditions.

Under light-starving conditions, only a sparse sequence of photon clicks can be detected over an extended period using a SPD, with each click represented as a “1” amid many “0 s” in the time series. The periodic nature of ${\widetilde{V}}_{{\rm{d}}}\left(t\right)$ allows reconstruction using photon-counting statistics. The delay time between the start signals and the photon clicks is statistically counted for each scan duration, initiated by the start signal. For an ideal periodic interference signal, when start signals are precisely provided, the scanning continues until the interferogram reaches the desired SNR (Fig. 1b). The data is then processed in a manner similar to classical DCS.

However, the interference signal can be affected by phase noise within the dual-comb system and time delays caused by optical path fluctuations. The dual-comb system’s phase noise affects the coherence and resolution of the down-converted RF comb (stability of ${\omega }_{0}$ and $\varDelta {f}_{{\rm{r}}}$), which can be mitigated using phase-locking techniques. Optical-path fluctuations, specified as ${r}_{{\rm{a}}}$, ${r}_{{\rm{d}}}$ and $\varDelta r$ in Eq. (1), contribute to random time delays and phase shifts in the interference signal. Particularly, the time delay caused by the varied $\varDelta r$ is amplified by a factor of $\frac{{f}_{{\rm{r}}}+\varDelta {f}_{{\rm{r}}}}{\varDelta {f}_{{\rm{r}}}}$, typically around ${10}^{6}$, presenting a significant challenge for the accurate reconstruction of dual-comb interference patterns through the photon-counting method. Such fluctuations behave as a specific issue for long-term photon-counting stability in fiber-based dual-comb systems due to fiber-length wandering^38^. The inherent variability in fiber length within the system is sufficient to disrupt the reconstruction of interference patterns (see Supplementary Fig. S9c), in addition to the unpredictable fluctuations from the sensing path.

It is observed that the dual-comb interference at output ‘c’ can be expressed as:

2 $${\widetilde{V}}_{{\rm{c}}}\left(t\right)=\mathop{\sum }\limits_{i=0}^{N-1}{\alpha }_{i}^{* }\cdot {\alpha }_{i}^{{\prime} }\,\cdot {e}^{j\cdot \left[\left(\varDelta \omega +i\cdot 2{\rm{\pi }}\varDelta {f}_{{\rm{r}}}\right)\left(t+\frac{{r}_{{\rm{c}}}+{r}_{{\rm{a}}}}{c}+\frac{{f}_{{\rm{r}}}+\varDelta {f}_{{\rm{r}}}}{\varDelta {f}_{{\rm{r}}}}\cdot \frac{\varDelta r}{c}\right)+\varOmega \right]}$$

Hence, the random jitter in the delay and phase shift, induced by variations in $\varDelta r$, ${r}_{{\rm{a}}}$ and $\varOmega$, remains consistent across ${\widetilde{V}}_{{\rm{c}}}\left(t\right)$ and ${\widetilde{V}}_{{\rm{d}}}\left(t\right)$. Leveraging this property, a trigger protocol can be implemented to enhance the stability of the photon-counting process. Temporal information of the interference pattern acquired at output ‘c’ in a classical mode can serve as the start signal for output ‘*d*’, effectively mitigating the random jitter. The remaining fluctuations, specifically those between *r*_c_ and ${r}_{{\rm{d}}}$, produce a time delay, reduced by the light speed $c$ as $\frac{{r}_{{\rm{c}}}-{r}_{{\rm{d}}}}{c}$. This is much smaller than one statistic bin required for photon counting, making it negligible even under the extreme external sensing path conditions (see Supplementary Note 2).

While this method applies to various dual-comb systems and SPDs, we use the all-fiber combs and free-running InGaAs SPDs, forming a compact polarization-maintaining all-fiber dual-comb interferometer (Fig. 1c), which is both portable and field-deployable. In addition, dividing a broad spectrum into segments and detecting them simultaneously with multiple single-photon detectors, referred to as segmented parallel detection, can significantly enhance the detection speed.

### Setup

**Fig. 2:**
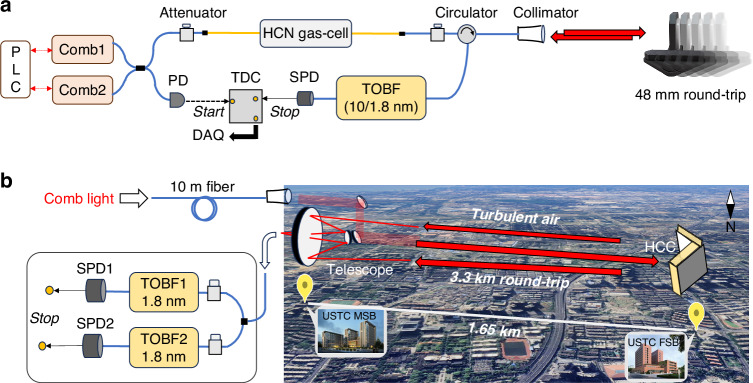
Experimental setup of the robust photon-counting dual-comb spectroscopy (DCS) under significant optical path fluctuations. PLC Phase-locking control unit, PD photon detector, TDC Time-to-digital converter, SPD single-photon detector, TOBF tunable optical bandpass filter, HCC hollow corner cube retroreflector. Optical attenuators reduce the detected power to the photon level, while tunable optical bandpass filters select measurement ranges of the target molecules. Blue and yellow solid lines represent the polarization-maintained (PM) single-mode fiber and the single-mode fiber, respectively. **a** In-lab photon-counting DCS. A moving mirror introduces artificial optical path fluctuations along the sensing path. Two types of TOBF are used for different demonstrations under attowatt-level light-starving conditions. The gas cell contains H^13^C^14^N at 25 Torr with a 16.5 cm path length. **b** Open-path photon-counting DCS. Two TOBFs with 1.8 nm bandwidths, centered at 1571 nm and 1573 nm, and two SPDs are used for segmented parallel detection of CO_2_, H_2_O, and HDO over a 3 km turbulent open-air path under light-starving conditions. The all PM-fiber-based system is placed in the laboratory, connected via two 10 m long PM fibers to an outdoor 3.3 km round-trip open-path link

The two self-referenced OFCs used in the present study are locked to a cavity-stabilized continuous-wave laser as the optical reference. The fiber-combined combs are directed to two separate output ports. One output is directly sent to a photodetector to generate a multiheterodyne beating signal, which is then threshold-triggered to produce the start signal for photon-counting statistics. The other output is used for photon-level DCS sensing. Our experiments include the HCN spectra detection under light-starving and fluctuating optical-path conditions, as well as open-path photon-counting DCS over a 3.3 km turbulent air path (Fig. 2).

### Attowatt-level broadband DCS under optical path fluctuations

**Fig. 3:**
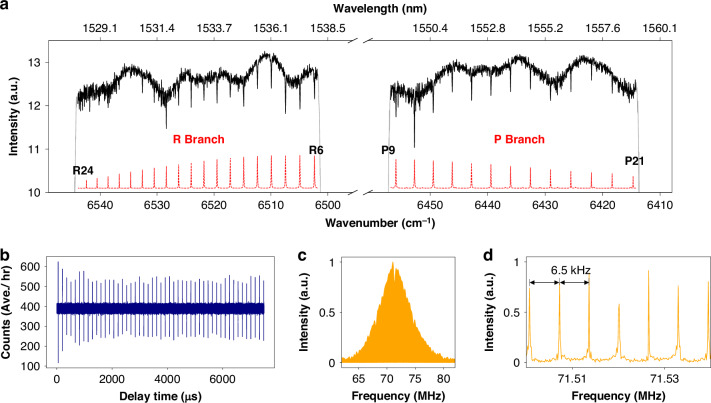
Photon-counting DCS at attowatt-level detection power per-comb line under severe optical path fluctuations conditions. **a** Broadband transmittance spectrum is obtained with an average detection power of 4 attowatts per-comb line after 10 h of statistical accumulation. The measurement covers the R-branch lines (R6–R24) at 1528–1538 nm and the P-branch lines (P9–P21) at 1548–1558 nm. The red dashed lines represent the calculated absorbance of H^13^C^14^N based on the HITRAN2020 database. **b** Photon-level interferogram (48 central fringes) demonstrates the comb-line-resolving capability. **c** Fourier transform analysis of the photon-level interferogram. The dips in the RF spectrum correspond to the absorption of H^13^C^14^N. **d** Enlarged view shows seven equally spaced comb lines with an interval of 6.5 kHz and the FWHM linewidth of 140 Hz

We demonstrate photon-counting DCS with a broad spectral bandwidth and attowatt-level detection power per-comb line. Besides the internal optical-path fluctuations from uncontrolled long-fiber drift, external fluctuations along the sensing path are simulated by a moving mirror with a 48 mm round-trip at 4 mm·s^−1^ (Fig. 2a). Due to the lack of broadband optical bandpass filters for segmented parallel detection as depicted in Fig. 1c, we use a 10 nm flat-top tunable optical filter to measure a 20 nm broadband spectrum. By tuning the center bandpass, we detect two spectral segments, 1548–1558 nm and 1528–1538 nm, using one InGaAs SPD in two sequential phases. The count rate of the SPD is set at 147.5 k·s^−1^, capturing one click per thousand laser pulses on average, with only 50 fW power detected before the SPD and a per-comb-line average power of 4 aW (see Supplementary Table S1). This is nearly ${10}^{10}$ times weaker than typical classical DCS optical power. After 10 h of accumulation for each phase, Fig. 3a presents the photon-counting transmittance spectra of H^13^C^14^N, showing the P- and R-branch absorption lines in the 2ν_3_ band.

To evaluate the comb-line-resolved spectrum, we extended the scanning time to exceed one interferogram period. To reduce the accumulation time, we replace the 10 nm filter with a Gaussian-shaped optical filter (1.8 nm FWHM, centered at 1551.3 nm) and set the count rate of the SPD to be 162 k·s^−1^, corresponding to an average detection power of 18.5 aW per-comb line before the SPD. Other experimental setups remain unchanged. This setup produces photon-level interferograms with a 7500 μs scan time, featuring 48 center fringes (Fig. 3b). The RF spectrum (Fig. 3c) derived from the Fourier transform of the interferograms distinctly exhibits the comb lines at ${\varDelta f}_{{\rm{r}}}$ intervals (Fig. 3d). The linewidth of these RF comb lines is approximately 140 Hz, matching the Fourier transform limit of the interferogram.

The SNR of the photon-level interferogram scales with the square root of the SPD’s count rate, even under extreme light-starving conditions where only a few femtowatts are detected by the SPD (Supplementary Fig. S1a). In particular, the SNR of the photon-level spectrum aligns closely with the theoretical shot-noise-limited SNR (see Supplementary Table S1). The SNR of the photon-level interferogram increases with the square root of the accumulation time, with a proportional constant of 0.5 (Supplementary Fig. S1b). This coherent addition pattern persists for up to 65 h under severe optical path jitter, limited by our acquisition duration. Theoretically, the SNR of the photon-counting DCS is proportional to the square root of the counting rate and accumulation time, while inversely proportional to the detected optical bandwidth (see Supplementary Note 3). Therefore, achieving high-SNR broadband spectra with attowatt detection power per-comb mode requires extended accumulation times.

**Fig. 4:**
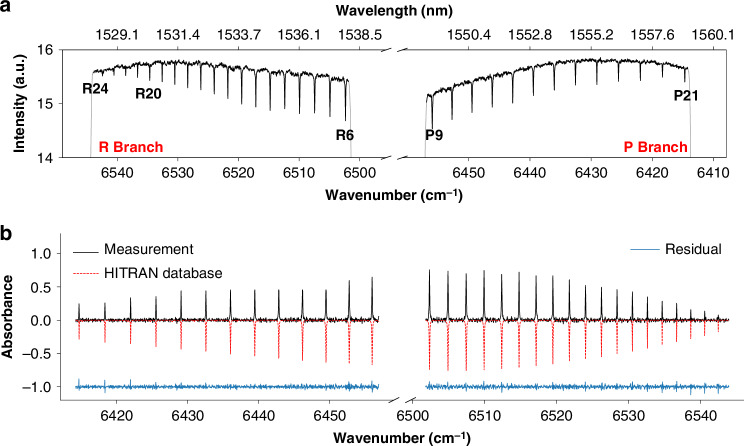
Broadband photon-counting DCS at an average detection power below 130 aW per-comb line. **a** Transmittance spectrum after 10 h of accumulation. **b** Comparison of the measured absorbance spectrum (black solid lines) with the HITRAN database (red dashed lines, inverted for clarity). The residual (blue solid line) between the experimental and theoretical spectra is also plotted in the same figure (shifted by −1 for clarity)

These experiments demonstrate the method’s viability at extremely low detection power levels. The resulting 20 nm broadband absorbance, extracted from the transmittance spectrum in Fig. 3a or even after longer accumulation time, shows relatively low SNR and a discrepancy from the database (Supplementary Fig. S2). Then we verify the broadband photon-counting DCS with a higher counting rate of 4.3 M·s^−1^, covering 8 interferogram periods and producing clearer center fringes, indicating comb-line resolution (Supplementary Fig. S3). Using the same setup as in Fig. 3a, including two sequential measurements for the P- and R-branch of H^13^C^14^N, the total power detected before the SPD is about 500 fW, or 120 aW per-comb line. Figure 4a presents a 20 nm broadband transmittance spectrum after a 10-h accumulation for each measurement phase. Compared to Fig. 3a, the spectral SNR is higher, revealing the absorption features (R20–R24, and P21) previously obscured by baseline noise. The absorption spectrum is in good agreement with the spectral database (Fig. 4b).

### Open-path photon-counting DCS with segmented parallel detection

We demonstrate the open-path photon-counting DCS using our proposed system (Fig. 2b), a promising technique for challenging remote sensing tasks. For fast atmospheric monitoring, the segmented parallel detection with a narrow bandwidth assigned to each SPD could reduce accumulation time by at least $\sqrt{{N}_{{\rm{s}}}}$ or even beyond ${N}_{{\rm{s}}}$-fold, where ${N}_{{\rm{s}}}$ is the number of SPDs (see “Methods”). Lacking the spectral division multiplexer component shown in Fig. 1c, we used a fiber beam splitter and two 1.8 nm tunable optical filters as a proof of concept.

The 3.3 km round-trip open-path link, traversing a traffic system and dense buildings within 50 m distance of the ground in an east-west orientation, experiences turbulence, complex environments, and direct afternoon sunlight. The combined combs emit 3 mW, and attenuators adjust the SPD count rates below 4.3 M·s^−1^ (about 550 fW), achieving a 93 dB open-path DCS attenuation. An observation campaign that lasted for more than 20 h confirms the system’s robustness against atmospheric turbulence and sunlight.

**Fig. 5:**
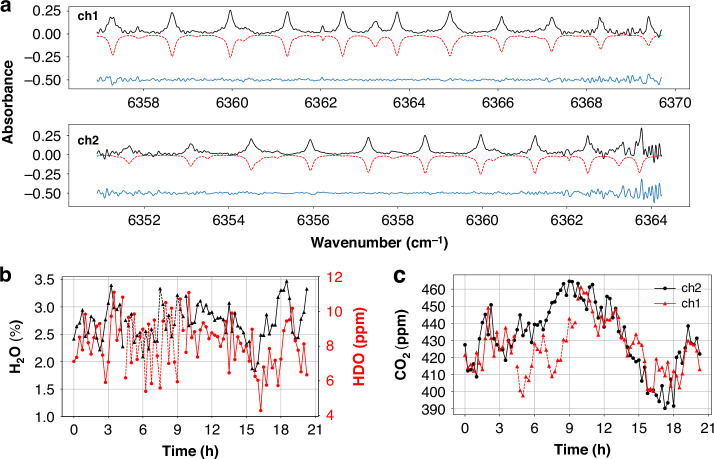
Open-path photon-counting DCS over a 24-h observation period. The observation started on 06/09/2024 at 21:00 (local time, UT + 8), marked as the zero point on the time axis. **a** Absorbance from atmospheric trace gases of ch1 and ch2 (black solid lines), and the calculated spectrum using the HITRAN2020 database for comparison (red dashed lines, inverted for clarity). The residual of them is presented in the blue line, shifted by -0.5 for clarity. The increased residual observed at the spectrum’s far-wing regions is attributed to the reduced power intensity in the vicinity of the Gaussian filter edges, which can be mitigated by using a flat-top filter. The results are accumulated over 3 h from 10:05 to 13:05 on 09/07. **b** Real-time water concentration with 15 min time-resolution extracted from ch1. H_2_O and HDO are plotted in units of percentage and ppm, represented by the black and red solid lines, respectively. **c** Real-time CO_2_ concentrations with 15 min time-resolution, retrieved from both ch1 and ch2, and plotted with red and black solid lines, respectively. The concentration data of ch1 retrieved when the SPD was over-saturated (about 4–9.5 h) are also included and represented by dashed lines in **b**, **c**

The two filters are centered at 1571.5 and 1573 nm (hereafter “ch1” and “ch2”). Figure 5a shows the absorbance from atmospheric trace gases after 3 h of accumulation, extracted from transmittance spectra segments of ch1 and ch2 (Supplementary Fig. S4a). In addition to the evenly spaced absorption lines of CO_2_, irregular line structures correspond to H_2_O and HDO absorption. Using the HITRAN2020 database for spectral analysis, the extracted spectra closely match the calculated spectra, though residuals are higher at the filter edges due to lower power.

Despite fluctuating telescope efficiency with environmental changes (Supplementary Fig. S4b), the system provided continuous real-time observations with a 15-min resolution. Diurnal CO_2_, H_2_O, and HDO concentrations were retrieved by fitting the absorbance to the HITRAN2020 database. Two strong absorption lines at 6362.05 cm^−1^ and 6363.24 cm^−1^, offering relatively high spectral SNR in ch1, are suitable for concentration retrieval. Figure 5b shows the concentrations of H_2_O and HDO retrieved from ch1, while Fig. 5c shows the concentration of CO_2_ from both channels. The telescope’s efficiency improved 4 h after the observation started due to favorable atmospheric conditions, leading to the saturation of the SPD in ch1 (highlighted in Supplementary Fig. S4b). The over-saturated SPD no longer operated in the photon-counting mode, rendering the results invalid. A tunable optical attenuator can be utilized for adjusting the SPD’s counting rate in future measurement campaigns to prevent the saturation issue. Apart from this period, the retrieved concentrations from the two channels are in good agreement, even under direct sunlight in the afternoon.

## Discussion

The proposed start-trigger protocol underscores the synchronization of common-mode interference patterns at the two outputs after the combination of two OFCs, effectively mitigating intractable internal path fluctuations. As the two OFCs traverse the external path in common mode, the fluctuations in the sensing path can be significantly suppressed. Compared to other photon-counting configurations (Supplementary Note 4), our approach offers practical sensing capabilities and enhanced stability in coherent photon-counting statistics. Our method surpasses the capabilities and applications of traditional DCS, achieving groundbreaking detection sensitivity at the attowatt-level per frequency comb mode. Remarkably, it preserves the high resolution and accuracy of traditional DCS, even under challenging measurement conditions. This property is crucial for integrating with existing DCS methods and substantially overcoming their limitations. The method achieves an SNR constrained only by shot noise (Table S1). In contrast, it is challenging for traditional DCS to reach the shot-noise limit due to factors like relative intensity noises (RIN), limited detector dynamic range and detector noise^39^. The long-term coherent accumulation ensures stable and high-SNR detection for the approach.

The comb-line resolution of the photon-counting spectrum is determined by the mutual coherence of the dual-comb. In this work, the comb-line-resolved spectrum is obtained by using the cavity-stabilized narrow-linewidth CW laser as the optical reference. Although the use of the cavity-stabilized laser in the system limits further field deployment, this issue can be addressed by replacing the cavity-stabilized laser with a free-running diode laser and using the “bootstrapped” frequency referencing scheme^40^. It should be noted that the dual-comb source with the RF reference scheme can also be deployed for photon-counting DCS. However, this phase-locking scheme (Supplementary Fig. S5) produces only an initial central burst fringe in the interferogram due to the low mutual coherence of the dual-comb source, which obscures the comb-line resolution.

Due to city regulations and permit requirements, our open-path photon-counting DCS was conducted over a 3.3 km open-path link in downtown Hefei, the capital of Anhui Province. Our system has successfully demonstrated open-path DCS that overcomes up to 93 dB attenuation with only several milliwatts of comb light, and even under complex conditions such as rain, fog, and seismic events (see Supplementary Fig. S10). This enables fieldable open-path DCS over distances exceeding 100 km using low optical power and compact telescopes with small apertures^41^. In a 100-km near-surface horizontal atmosphere, optical delay fluctuations caused by atmospheric turbulence generally remain within a few nanoseconds^42^. Since reconstructing the photon-level interferogram only requires a time resolution of $1/{f}_{{\rm{r}}}$ for photon clicks statistics, such time-delay jitter is generally permissible and does not impact the accuracy of the interference signal (see Supplementary Note 2.1). In other words, atmospheric turbulence over such distances does not hinder the system’s performance. Recently, a 113-km open-path DCS system demonstrated the feasibility of large-scale greenhouse gas monitoring networks^9^. However, that system relied on watt-level high-power comb sources and complex configurations, posing challenges in terms of cost, operability, eye safety, and spectral bandwidth. In contrast, our method provides a promising and practical solution for building this monitoring network more effectively.

We demonstrated high-sensitivity and broadband photon-counting DCS using a common-mode configuration with a start-signal triggered protocol. Even under significant optical path fluctuations, the approach exhibits comb-line resolution and shot-noise-limited SNR of the spectrum. This study also marks the first demonstration of atmospheric open-path photon-counting DCS, which was realized in a compact, all-fiber setup supporting energy efficiency and eye safety. Looking ahead, this technology holds great promise for high-precision DCS in challenging environments such as turbulent open-air conditions or flowing bodies of water, where significant optical path fluctuations and energy attenuation are typical. This advancement paves the way for quantifying trace gases in a variety of dynamic and appealing sensing applications, from monitoring non-cooperative targets to achieving extensive sensing ranges over hundreds of kilometers, and even establishing open-path links from the ground to satellites.

## Methods

### Dual-comb light source

Two homemade erbium-doped fiber mode-locked femtosecond lasers, each operating at a 200 MHz repetition rate, are used as the dual-comb source. Each OFC emits 4 ${\rm{mW}}$ average power, centered at 1550 nm, with a spectral range of 100 nm. The carrier-envelope offset frequency (${f}_{{\rm{ceo}}}$) of each laser is stabilized by the f-2f self-referencing technique, and both RF reference and optical reference schemes can be used to stabilize the repetition frequency (*f*_r_) (Supplementary Fig. S6a). A homemade vacuum-cavity-stabilized laser, with a central frequency of 193.4 THz and Hz-level linewidth, is used here as the optical reference. All radio frequencies used for phase-locking are traced to an Rb atomic clock (SRS FS725). In this study, we use the optical reference scheme for the dual-comb source to achieve high mutual coherence.

### Photon-counting interferometer

The all-fiber photon-counting interferometer is built based on the dual-comb source shown in Supplementary Fig. S6b. The two OFCs are combined with a fiber coupler, with one output attenuated to about 10 μW average power and subsequently detected by a photodetector (Thorlabs PDB570C). To satisfy the Nyquist sampling condition, a fiber-coupled tunable optical filter (WL Photonics) with a 1.8 nm FWHM is used. An electronic low-pass filter is used to ensure the one-to-one mapping of comb lines to the RF domain. The central fringes of the resulting interferogram are threshold-triggered. The triggered timestamps serve as the start signal. The other output from the fiber coupler is used for photon-level detection. InGaAs SPDs (Quantum-CTek, QCD600B-H) with a quantum efficiency of 35.5% are used to capture photons along the sensing path. The after-pulsing probability and dark count rate are approximately 1% and below 2 k·s^−1^, respectively. A time-to-digital converter (TDC) calculates the delay between the start signal and photon clicks. The time resolution of the TDC is set to 500 ps, and a digital low-pass filter is applied to the raw photon-counting data to retrieve the photon-level interferogram (Supplementary Fig. S6c).

### Segmented parallel detection

The spectral signal-to-noise ratio for a single SPD-based photon-counting DCS can be expressed as (see Supplementary Note 3):

M.1 $${\left(\frac{S}{N}\right)}_{{\rm{\nu }}}=\sqrt{2}\cdot \frac{1}{M}\cdot \frac{V}{\sqrt{1+V}}\cdot \sqrt{{N}_{{\rm{CR}}}\cdot {T}_{{\rm{eff}}}}$$

Where $M$ is the number of comb teeth, *V* is the interference visibility, ${N}_{{CR}}$ is the count rate, and *T*_*eff*_ is the integration time. For simplicity, without loss of generality, we consider a special type of segmented parallel detection: the broadband flat-top spectrum is divided into ${N}_{s}$ segments with an equal optical bandwidth, and each segment is detected by one SPD. Meanwhile, it is assumed that the interference visibility $V$ remains consistent with that of the original spectrum across each spectral segment. The detection of the original broadband spectrum using a single SPD is referred to as the original detection.

*Case 1*: When the detection power of each segment is lower than the saturation count rate of the SPD (denoted as ${N}_{{\rm{SCR}}}$ hereafter), the count rate of each single-photon detector is ${N}_{{\rm{CR}}}/{N}_{{\rm{s}}}$. Based on Eq. (M.1), the SNR of the piece spectrum, ${{\left(\frac{S}{N}\right)}^{{\prime} }}_{{\rm{\nu }}}$, with the same accumulation time *T*_eff_ is:

M.2 $${{\left(\frac{S}{N}\right)}^{{\prime} }}_{{\rm{\nu }}}=\sqrt{{N}_{{\rm{s}}}}\cdot {\left(\frac{S}{N}\right)}_{{\rm{\nu }}}$$

Hence, the detection time for the segmented parallel detection can be reduced by a factor of $\sqrt{{N}_{s}}$ compared to the original detection.

*Case 2:* When the detection power of some segments is beyond ${N}_{{\rm{SCR}}}$, additional optical attenuation should be deployed before the SPDs. It is worth noting that the additional optical attenuation is also required for the original detection, and the maximal count rate ${N}_{{CR}}$ is limited to ${N}_{{\rm{SCR}}}$, or ${N}_{{\rm{CR}}}\le {N}_{{\rm{SCR}}}$. In segmented parallel detection, the count rate of each SPD equals *N*_CR_ or even exceeds this value, constrained by ${N}_{{\rm{SCR}}}$:

M.3 $$\sqrt{\frac{{N}_{{\rm{SCR}}}}{{N}_{{\rm{CR}}}}}{N}_{{\rm{s}}}\cdot {\left(\frac{S}{N}\right)}_{{\rm{\nu }}}\ge {{\left(\frac{S}{N}\right)}^{{\prime} }}_{{\rm{\nu }}}\ge {N}_{{\rm{s}}}\cdot {\left(\frac{S}{N}\right)}_{{\rm{\nu }}}$$

Therefore, the detection time for the segmented parallel detection can be reduced by a factor of at least ${N}_{{\rm{s}}}$.

In summary, this segmented parallel detection method can enhance the speed by at least a factor of $\sqrt{{N}_{s}}$. In certain scenarios, this enhancement can reach or even exceed a factor of $\sqrt{{N}_{{\rm{s}}}}$.

## Supplementary information

Supplementary Information for: Broadband photon-counting dual-comb spectroscopy with attowatt sensitivity over turbulent optical paths

## Data Availability

All data supporting the main findings of this work are available within the paper and its Supplementary Information, or available from the corresponding author upon reasonable request.
