# Supplementary material for: Broadband photon-counting dual-comb spectroscopy with attowatt sensitivity over turbulent optical paths
Source: Light Sci Appl. 2025 Aug 27;14:293. doi: 10.1038/s41377-025-01934-7 (PMC12381157; doi:10.1038/s41377-025-01934-7)
Supplement: Supplementary file 1 — Supplementary Information for: Broadband photon-counting dual-comb spectroscopy with attowatt sensitivity over turbulent optical paths [file 41377_2025_1934_MOESM1_ESM.pdf]

Supplementary Information for:

Broadband photon-counting dual-comb spectroscopy with attowatt sensitivity over turbulent optical paths

Wei Zhong[1,6], Yingyu Liu[1], Qin Yin[1], Ruocan Zhao[1*], Chong Wang[1], Wei Ren[2*], Xiankang Dou[3] & Xianghui Xue [1,3,4,5*]

[1]CAS Key Laboratory of Geospace Environment, School of Earth and Space Sciences, University of Science and Technology of China, Hefei 230026, China

[2]Department of Mechanical and Automation Engineering, The Chinese University of Hong Kong, New Territories, Hong Kong SAR, China

[3]Hefei National Laboratory, University of Science and Technology of China, Hefei 230088, China

[4]Hefei National Research Center for Physical Sciences at the Microscale and School of Physical Sciences, University of Science and Technology of China, Hefei 230026, China

[5]CAS Center for Excellence in Comparative Planetology, Anhui Mengcheng Geophysics National Observation and Research Station, University of Science and Technology of China, Hefei 230026, China

[6]Present address: Department of Mechanical and Automation Engineering, The Chinese University of Hong Kong, New Territories, Hong Kong SAR, China

*Corresponding authors

Email address and contact details:
Wei Zhong: zwei19@mail.ustc.edu.cn , weizhong@cuhk.edu.hk
Yingyu Liu: liuyingyu@mail.ustc.edu.cn
Qin Yin: yinqin@mail.ustc.edu.cn
Ruocan Zhao: canlan@ustc.edu.cn
Chong Wang: wclhy50@ustc.edu.cn
Wei Ren: renwei@mae.cuhk.edu.hk
Xiankang Dou: dou@ustc.edu.cn
Xianghui Xue: xuexh@ustc.edu.cn, (Tel: +86-551-63600048)

Note 1. Theory of dual-comb single photon interference

As depicted in Fig. 1a, the field operators in the four paths (a, b, c, and d, with path length $\vec{r}_{\mathrm{a}}$, $\vec{r}_{\mathrm{b}}$, $\vec{r}_{\mathrm{c}}$, and $\vec{r}_{\mathrm{d}}$, respectively) can be interrelated as:

$$\begin{pmatrix} \hat{E}_{\mathrm{c}} \\ \hat{E}_{\mathrm{d}} \end{pmatrix} = \begin{pmatrix} \sqrt{T} & j\cdot\sqrt{R} \\ j\cdot\sqrt{R} & \sqrt{T} \end{pmatrix} \cdot \begin{pmatrix} \hat{E}_{\mathrm{a}} \\ \hat{E}_{\mathrm{b}} \end{pmatrix} \tag{S.1}$$

where T and R are the transmission and reflection coefficients of the beam splitter, respectively. According to Eq. (S.1), the filed operators $\hat{E}_{\mathrm{c}}$ and $\hat{E}_{\mathrm{d}}$ before reaching the PD and SPD can be written in the Heisenberg picture as:

$$\hat{E}_{\mathrm{c}} = \hat{E}_{\mathrm{c}}^{(+)} + \hat{E}_{\mathrm{c}}^{(-)}$$

$$= \sqrt{T}\sum_{i=0}^{N-1} \hat{a}_i \cdot e^{j\cdot[\omega_i\cdot t + \vec{k}_i\cdot(\vec{r}_a+\vec{r}_c)]} + j\cdot\sqrt{R}\sum_{i=0}^{N-1} \hat{a}'_i \cdot e^{j\cdot[\omega'_i\cdot t + \vec{k}'_i\cdot(\vec{r}_b+\vec{r}_c)]} + H.C. \tag{S.2}$$

$$\hat{E}_d = \hat{E}_{\mathrm{d}}^{(+)} + \hat{E}_{\mathrm{d}}^{(-)}$$

$$= j\cdot\sqrt{R}\sum_{i=0}^{N-1} \hat{a}_i \cdot e^{-\chi_i} \cdot e^{j\cdot[\omega_i\cdot t + \vec{k}_i\cdot(\vec{r}_{\mathrm{a}}+\vec{r}_{\mathrm{d}})]} + \sqrt{T}\sum_{i=0}^{N-1} \hat{a}'_i \cdot e^{-\chi'_i} \cdot e^{j\cdot[\omega'_i\cdot t + \vec{k}'_i\cdot(\vec{r}_{\mathrm{b}}+\vec{r}_{\mathrm{d}})]} + H.C. \tag{S.3}$$

where $\hat{E}_{\mathrm{c(d)}}^{(+)}$ and $\hat{E}_{\mathrm{c(d)}}^{(-)}$ are Hermit conjugates of each other, referred to the positive frequency operator and negative frequency operator, respectively. The vectors $\vec{k}_i$ and $\vec{k}'_i$ represent the wave vectors of the states $|\alpha_i\rangle$ and $|\alpha_i'\rangle$, with values ω_i/c and ω'_i/c, respectively. The parameters χ_i or χ'_i are complex, with the imaginary part representing the phase response and the real part representing attenuation along the paths. The annihilation operators $\hat{a}_i$ and $\hat{a}'_i$ are the eigenoperators for coherent state $|\alpha_i\rangle$ and $|\alpha_i'\rangle$ and satisfy:

$$\hat{a}_i^{(\prime)} \left|\alpha_k^{(\prime)}\right\rangle = \begin{cases} \alpha_i^{(\prime)} \left|\alpha_i^{(\prime)}\right\rangle, & i = k \\ 0, & i \neq k \end{cases} \tag{S.4}$$

where α_i represents the eigenvalue of the state $|\alpha_i\rangle$, and $|\alpha_i|^2$ is the statistical expected photon number of state $|\alpha_i\rangle$.

Based on the above, the detection probability $P_{\mathrm{c}}(t)$ on the PD is given by:

$$P_{\mathrm{c}}(t) = \langle\psi_{\mathrm{a}}|\langle\psi_{\mathrm{b}}|\hat{E}_{\mathrm{c}}^{(-)}\hat{E}_{\mathrm{c}}^{(+)}|\psi_{\mathrm{b}}\rangle|\psi_{\mathrm{a}}\rangle$$

$$= \left(\hat{E}_{\mathrm{c}}^{(+)}|\psi_{\mathrm{b}}\rangle|\psi_{\mathrm{a}}\rangle\right)^{\dagger} \cdot \hat{E}_{\mathrm{c}}^{(+)}|\psi_{\mathrm{b}}\rangle|\psi_{\mathrm{a}}\rangle$$

$$= \left(\sqrt{T}A_{\mathrm{c1}} + j\sqrt{R}A_{\mathrm{c2}}\right)^{*}(\sqrt{T}A_{\mathrm{c1}} + j\sqrt{R}A_{\mathrm{c2}})\langle\psi_{\mathrm{a}}|\langle\psi_{\mathrm{b}}||\psi_{\mathrm{b}}\rangle|\psi_{\mathrm{a}}\rangle$$

$$= T(A_{c1}^* \cdot A_{c1}) + R(A_{c2}^* \cdot A_{c2}) - 2\sqrt{RT} \cdot Im\{A_{c1}^* \cdot A_{c2}\} \tag{S.5}$$

where,

$$A_{c1}^* = \sum_{i=0}^{N-1} \alpha_i \cdot e^{j \cdot [\omega_i \cdot t + \vec{k}_i \cdot (\vec{r}_a + \vec{r}_c)]} \tag{S.6}$$

$$A_{c2}^* = \sum_{i=0}^{N-1} \alpha_i' \cdot e^{j \cdot [\omega_i' \cdot t + \vec{k}'_i \cdot (\vec{r}_b + \vec{r}_d)]} \tag{S.7}$$

DCS only considers the low-frequency term below $f_r/2$ to ensure the one-to-one mapping relationship from the optical spectrum to the RF spectrum, which is realized by employing an electronic or digital $f_r/2$ low-pass filter. Considering the one-to-one mapping relationship, $P_c(t)$ can be written as:

$$P_c(t) = T \sum_{i=0}^{N-1} \alpha_i^* \cdot \alpha_i + R \sum_{i=0}^{N-1} \alpha'^*_i \cdot \alpha_i' - 2\sqrt{RT} \cdot Im\{\tilde{V}_c(t)\} \tag{S.8}$$

where the first two terms are the direct content, and $\tilde{V}_c(t)$ represents the interference term. Denoting $\Delta\vec{r} = \vec{r}_b - \vec{r}_a$, $\omega_0 = \omega_b - \omega_a$ and $\Omega = \frac{\Delta r}{c} \cdot (\omega_b - \frac{f_r + \Delta f_r}{\Delta f_r} \cdot \omega_0)$, $\tilde{V}_c(t)$ can be detailed as:

$$\tilde{V}_c(t) = \sum_{i=0}^{N-1} \alpha_i^* \cdot \alpha_i' \cdot e^{j \cdot [(\omega_i' - \omega_i) \cdot t + (\vec{k}'_i - \vec{k}_i) \cdot \vec{r}_c + \vec{k}'_i \cdot \vec{r}_b - \vec{k}_i \cdot \vec{r}_a]}$$

$$= \sum_{i=0}^{N-1} \alpha_i^* \cdot \alpha_i' \cdot e^{j \cdot \left[(\Delta\omega + i \cdot 2\pi \Delta f_r)\left(t + \frac{r_c + r_a}{c}\right) + \vec{k}'_i \cdot \Delta\vec{r}\right]}$$

$$= \sum_{i=0}^{N-1} \alpha_i^* \cdot \alpha_i' \cdot e^{j \cdot \left[(\Delta\omega + i \cdot 2\pi \Delta f_r)\left(t + \frac{r_c + r_a}{c} + \frac{f_r + \Delta f_r}{\Delta f_r} \frac{\Delta r}{c}\right) + \Omega\right]} \tag{S.9}$$

Similarly, $P_d(t)$ can be written as:

$$P_d(t) = R \sum_{i=0}^{N-1} \alpha_i^* \cdot \alpha_i \cdot e^{-(\chi_i^* + \chi_i)} + T \sum_{i=0}^{N-1} \alpha'^*_i \cdot \alpha_i' \cdot e^{-(\chi'^*_i + \chi_i')} + 2\sqrt{RT} \cdot Im\{\tilde{V}_d(t)\} \tag{S.10}$$

The interference term $\tilde{V}_d(t)$ is detailed as follows:

$$\tilde{V}_d(t) = \sum_{i=0}^{N-1} \alpha_i^* \cdot \alpha_i' \cdot e^{-(\chi_i^* + \chi_i')} \cdot e^{j \cdot \left[(\Delta\omega + i \cdot 2\pi \Delta f_r)\left(t + \frac{r_d + r_a}{c} + \frac{f_r + \Delta f_r}{\Delta f_r} \frac{\Delta r}{c}\right) + \Omega\right]} \tag{S.11}$$

Here, Eq. (S.11) and Eq. (S.8) correspond to Eq. (3) and Eq. (4) in the main text, respectively.

Note 2. Discussion of the start signal-triggered photon-counting protocol

The interference term $Im\{\tilde{V}_{\mathrm{c}}(t)\}$ in the time domain can be expressed as a sum of a series of carrier-envelope signals:

$$V_{\mathrm{c}}(t) = \sum_{n=1}^{+\infty} A'(t - T'_n) \cdot cos[\omega_{\mathrm{c}}(t - T'_n) + \Omega + n \cdot \Delta\varphi_{\mathrm{ceo}}] \tag{S.12}$$

$$T'_n = n\frac{1}{\Delta f_{\mathrm{r}}} + \delta t_n^{(\mathrm{c})} + \delta t_n^{(\mathrm{a})} + \frac{f_{\mathrm{r}} + \Delta f_{\mathrm{r}}}{\Delta f_{\mathrm{r}}} \cdot \delta t_n^{(\Delta r)} \tag{S.13}$$

where ω_{c} is the carrier's central frequency, and $\Delta\varphi_{\mathrm{ceo}}$ is the carrier-envelope phase offset; T'_n is the arrival time of the nth carrier-envelope signal in the time domain; and $\delta t_n^{(\mathrm{c})}$, $\delta t_n^{(\mathrm{a})}$, and $\delta t_n^{(\Delta r)}$ represent the time delay of light for the nth carrier-envelope signal, caused by path length variations of $r_{\mathrm{c}}, r_{\mathrm{a}}$, or Δr, respectively. Similarly, the probability amplitude of photon detection for single-photon dual-comb interference, $Im\{\tilde{V}_{\mathrm{d}}(t)\}$, can be expressed as:

$$V_{\mathrm{d}}(t) = \sum_{n=1}^{+\infty} A(t - T_n) \cdot cos[\omega_{\mathrm{c}}(t - T_n) + \Omega + n \cdot \Delta\varphi_{\mathrm{ceo}}] \tag{S.14}$$

$$T_n = n\frac{1}{\Delta f_{\mathrm{r}}} + \delta t_n^{(\mathrm{d})} + \delta t_n^{(\mathrm{a})} + \frac{f_{\mathrm{r}} + \Delta f_{\mathrm{r}}}{\Delta f_{\mathrm{r}}} \cdot \delta t_n^{(\Delta r)} \tag{S.15}$$

where $\delta t_n^{(\mathrm{d})}$ is the time delay of light caused by path length variations of d. Besides, we denote $\delta t_n^{\mathrm{trig}}$ as the timing error of the start signal for the nth carrier-envelope signal, which depends on the precision of threshold-triggering. Therefore, the total timing error in the proposed start signal-triggered photon-counting protocol for the nth carrier-envelope signal, denoted as ΔT_n, is given by:

$$\Delta T_n = \delta t_n^{\mathrm{trig}} + T_n - T'_n = \delta t_n^{\mathrm{trig}} + \delta t_n^{(\mathrm{d})} - \delta t_n^{(\mathrm{c})} \tag{S.16}$$

Therefore, after photon-counting statistics, the reconstructed single-photon interference possibility, denoted as $I_{\mathrm{pc}}(\tau)$, can be written as:

$$I_{\mathrm{pc}}(\tau) = \sum_{n=1}^{K} A(\tau - \Delta T_n) \cdot cos[\omega_{\mathrm{c}}(\tau - \Delta T_n) + n \cdot \Delta\varphi_{\mathrm{ceo}}] \tag{S.17}$$

where the number of K reflects the duration of the accumulation. Next, based on Eq. (S.17), we discuss the impact of ΔT_n and non-zero carrier-envelope phase offset on the results of photon-counting DCS.

(2.1) Impact of path-length fluctuations

Firstly, we discuss the impact of the trems $\delta t_n^{(\mathrm{d})}$ and $\delta t_n^{(\mathrm{c})}$ in Eq. (S.16), which is equal to

$\delta r_{\mathrm{d}}/c$ and $\delta r_{\mathrm{d}}/c$, respectively. In photon-counting DCS, the demanded time resolution to record the time delay between the start signals and the photon clicks, or the time length of a statistical bin, is $\frac{1}{f_{\mathrm{r}}}$. For example, the OFCs used in the text are with 200 MHz repetition rates, and the time length of a statistical bin is thus 5 ns.

In practical applications, δr_{c} corresponds to the wandering of a small segment of fiber, which is typically on the micron scale. Therefore, $\delta t_n^{(\mathrm{c})}$ caused by the fiber-length wandering is on the femtosecond scale. Moreover, this wandering can be further suppressed by temperature and vibration control. The impact of δr_{c} on photon-counting statistics can thus be ignored. δr_{d} corresponds to uncontrollable fluctuations in the remote sensing path, such as turbulence in open-air detection. The impact of δr_{d} only becomes an issue on photon-counting statistics when $\delta r_{\mathrm{d}} > \frac{c}{f_{\mathrm{r}}}$, which corresponds to 1.5 m in our system. Measurements of the horizontal open-air path near the Earth's surface using dual-comb two-way time-frequency transfer (TWTFT) indicate that the total time delay drift over 113 km during 3 hours is less than 2 ns, and less than 0.1 ns during the stable periods (see Fig. 2.c in the ref. [42] of the text). Thus, even applying to open-path DCS over a hundred kilometers in a relatively normal atmosphere environment, δr_{d} induced time-delay typically remains within a statistical bin.

In summary, the impact of the term $\delta t_n^{(\mathrm{d})} - \delta t_n^{(\mathrm{c})}$ in ΔT_n, as depicted in Eq. (S16), can be considered negligible in photon-counting statistics.

(2.2) Influence of timing error in the start signal

Secondly, we discuss the impact if the timing error in the start signal, $\delta t_n^{\mathrm{trig}}$. When well-locked and highly coherent dual-comb sources are employed and the interference signal for threshold triggering exhibits a favorable signal-to-noise ratio (SNR), the timing errors induced by relative intensity noise and the precision of the electronic triggering, which is substantially smaller than a statistical bin, can be neglected. In this context, we mainly consider the timimg error in the start signals caused by the carrier-envelope phase shift when $\Delta\varphi_{\mathrm{ceo}}$ is non-zero. According to the characteristics of electronic leading-edge threshold triggering, the variation in the triggering time points is related to the slope of the envelope at the triggering position. The steeper the slope of the envelope, the smaller the variation in the triggering time points; but this variation ranges between

half and a full carrier-wave cycle, i.e., $(\frac{\pi}{\omega_c}, \frac{2\pi}{\omega_c})$. Meanwhile, the interference signal needs to satisfy the Nyquist sampling, with $\frac{\omega_c}{2\pi}$ generally around $f_r/4$. Hence, we estimate that δt_n^{trig} is on the order of tens of nanoseconds, corresponding to 2-4 statistical bins when $\Delta\varphi_{\text{ceo}}$ is non-zero.

(2.3) Impact of carrier-envelope phase offset

According to the discussion in Section (2.2), the timing error caused by δt_n^{trig} can exceed a statistical bin, which is unfavorable for the reconstruction of the single-photon interferogram. However, experimental results indicate that the successful reconstruction is not affected even when $\Delta\varphi_{\text{ceo}}$ is non-zero (Fig. S7). Now, we discuss the impact of $\Delta\varphi_{\text{ceo}}$ on the results of photon-counting DCS. The threshold-triggered process can be depicted as:

$$A'(t_n^{\text{trig}}) \cdot cos[\omega_c(t_n^{\text{trig}}) + n \cdot \Delta\varphi_{\text{ceo}}] = T_h \tag{S.18}$$

where t_n^{trig} is the timestamp of the nth triggering point and T_h is the threshold value of triggering. When $\Delta\varphi_{\text{ceo}} \neq 0$, t_n^{trig} varies with $n \cdot \Delta\varphi_{\text{ceo}}$ due to carrier-envelope shift. This shift results in a triggering timestamp jitter, denoted as δt_n^{trig}, where $t_n^{\text{trig}} = t_0^{\text{trig}} + \delta t_n^{\text{trig}}$. And $A'(t_n^{\text{trig}})$ can be expanded by the first-order approximation as:

$$A'(t_n^{\text{trig}}) = T_h + k_{\text{env}} \cdot \delta t_n^{\text{trig}} \tag{S.19}$$

where k_{env} is the slope of the envelope at the triggering threshold. Thus, we have:

$$cos[\omega_c t_n^{\text{trig}} + n \cdot \Delta\varphi_{\text{ceo}}] = \frac{1}{1 + \frac{k_{\text{env}}}{T_h} \cdot \delta t_n^{\text{trig}}} \approx 1 - \frac{k_{\text{env}}}{T_h} \cdot \delta t_n^{\text{trig}} \tag{S.20}$$

And we denote the phase term of the carrier signal as $\phi_n = \omega_c t_n^{\text{trig}} + n \cdot \Delta\varphi_{\text{ceo}}$. This implies that the value of $cos[\phi_n]$ is nearly 1. Thus, we can treat ϕ_n as zero. Combining the above discussions, Eq. (S.17) can be expressed as:

$$I_{\text{pc}}(\tau) = \sum_{n=1}^{K} A(\tau + t_n^{\text{trig}}) \cdot cos[\omega_c(\tau + t_n^{\text{trig}}) + n \cdot \Delta\varphi_{\text{ceo}}]$$
$$= \sum_{n=1}^{K} A(\tau + t_0^{\text{trig}} + \delta t_n^{\text{trig}}) \cdot cos[\omega_c \tau + \phi_n]$$
$$\approx \sum_{n=1}^{K} A(\tau + t_0^{\text{trig}} + \delta t_{\text{n}}^{\text{trig}}) \cdot cos[\omega_c \tau] \tag{S.21}$$

Eq. (S.21) implies that the impact of non-zero $\Delta\varphi_{\mathrm{ceo}}$ mainly presents as the jitter of the envelope. According to the discussion in (2.2), the standard of this jitter is approximately 2-4 statistical bins. The spectrum of the constructed photon-counting interferogram, denoted as $Sp(\nu)$, is given by the Fourier transform of Eq. (S.21):

$$Sp(\nu) = \mathcal{F}\{\sum_{n=1}^{K} A\left(\tau + t_0^{\mathrm{trig}} + \delta t_n^{\mathrm{trig}}\right) \cdot cos[\omega_c\tau]\}$$

$$= \sum_{n=1}^{K} G\left(\nu - \frac{\omega_c}{2\pi}\right) \cdot e^{i2\pi\nu\cdot(t_0^{\mathrm{trig}}+\delta t_n^{\mathrm{trig}})}$$

$$= G\left(\nu - \frac{\omega_c}{2\pi}\right) \cdot e^{i2\pi\nu t_0^{\mathrm{trig}}} \sum_{n=1}^{K} e^{i2\pi\nu\delta t_n^{\mathrm{trig}}} \tag{S.22}$$

Where $G\left(\nu - \frac{\omega_c}{2\pi}\right)$ is the RF spectrum, and $e^{i2\pi\nu t_0^{\mathrm{trig}}}$ is a fixed linear phase shift. Thus, our focus shifts to the summation term $\sum_{n=1}^{K} e^{i2\pi\nu\delta t_n^{\mathrm{trig}}}$. In photon-counting statistics, long-term accumulation (with a large (K)) is required for statistical analysis. $\sum_{n=1}^{K} e^{i2\pi\nu\delta t_n^{\mathrm{trig}}}$ can be considered as the expectation of the exponential random variable as $E\left[e^{i2\pi\nu\cdot X}\right]$ where $X \sim \delta t_n^{\mathrm{trig}}$.

Next, we demonstrate that $E\left[e^{i2\pi\nu\cdot X}\right]$ converges and is not identically zero. When $\Delta\varphi_{\mathrm{ceo}}$ is non-zero, $2\pi/\Delta\varphi_{\mathrm{ceo}}$ is divided into two cases, namely rational and irrational numbers, for discussion:

a) When $2\pi/\Delta\varphi_{\mathrm{ceo}}$ is a rational number, X is discretely uniformly distributed. Assuming there are m discrete values in X, we have:

$$E\left[e^{i2\pi\nu\cdot X}\right] = \frac{1}{m}\sum_{j=1}^{m} e^{i2\pi\nu\delta t_j^{\mathrm{trig}}} \tag{S.23}$$

b) When $2\pi/\Delta\varphi_{\mathrm{ceo}}$ is an irrational number, X can be considered as continuously uniformly distributed (with $\delta t_n^{\mathrm{trig}}$ uniformly distributed over the interval $[a, b]$ with probability density function $f_X(x) = \frac{1}{b-a}, a \le x \le b$). The expectation is given by:

$$E\left[e^{i2\pi\nu\cdot X}\right] = \int_a^b e^{i2\pi\nu x} \cdot f_X(x)dx$$

$$= \frac{1}{b-a}\int_a^b e^{i2\pi\nu x}\,dx$$

$$= \frac{e^{i2\pi \nu b} - e^{i2\pi \nu a}}{(b-a) \cdot i2\pi \nu} \quad (S.24)$$

The above analysis demonstrates that in this start signal-triggered photon-counting protocol, even with a non-zero $\Delta\varphi_{\mathrm{ceo}}$, the resulting photon-level spectrum $Sp(\nu)$, has a well-defined statistical meaning and converges. $Sp(\nu)$ includes the target spectral detection term and an additional low-frequency modulation term (as described by Eq. (S.23) or Eq. (S.24)). This additional modulation term corresponds to slow fluctuations of the spectral baseline but can generally be mitigated or removed during spectral inversion.

When $\Delta\varphi_{\mathrm{ceo}} = 0$, the start signal's timing error is negatable, as $\delta t_n^{\mathrm{trig}} = 0$ and $\Delta T_n = 0$. In this case, Eq. (S.17) simplifies to:

$$I_{\mathrm{pc}}(\tau) = \sum_{n=1}^{K} A(\tau) \cdot cos[\omega_{\mathrm{c}}\tau] \quad (S.25)$$

It is easy to prove that the accumulated photon-level spectrum $Sp(\nu)$ at this point corresponds to the desired target $G\left(\nu - \frac{\omega_{\mathrm{c}}}{2\pi}\right)$. By adjusting the locking scheme, it is relatively easy to achieve $\Delta f_{\mathrm{ceo}} = 0$ and $f_{\mathrm{r}}/\Delta f_{\mathrm{r}}$ as an integer, thereby achieving $\Delta\varphi_{\mathrm{ceo}} = 0$ and then obtaining a more "perfect" photon-level spectrum.

Note 3. Thermotical shot-noise-limit SNR for photon-counting DCS

The SNR of the photon-counting dual-comb interferogram at zero optical delay (t=0) is dominated by shot noise [21]:

$$\left(\frac{S}{N}\right)_{t=0} = \frac{n_{\mathrm{interf}}}{\sqrt{n + n_{\mathrm{interf}}}} \quad (S.26)$$

where n_{interf} is the number of photon counts from the maximum aptitude of the interference signal, and $(n + n_{\mathrm{interf}})$ represents the total number of photons counted at zero time-delay bin. According to the Poisson statistical characteristics, the corresponding shot noise is $\sqrt{n + n_{\mathrm{interf}}}$. The maximum and minimum photon counts are thus $n_{\mathrm{interf,\,max}} = n + n_{\mathrm{interf}}$ and $n_{\mathrm{interf,\,min}} = n - n_{\mathrm{interf}}$, respectively. The interference visibility V is defined as:

$$V = \frac{n_{\mathrm{interf,\,max}} - n_{\mathrm{interf,\,min}}}{n_{\mathrm{interf,\,max}} + n_{\mathrm{interf,\,min}}} = \frac{n_{\mathrm{interf}}}{n} \quad (S.27)$$

The SNR in the frequency domain at a specific frequency ν is related to $\left(\frac{S}{N}\right)_{t=0}$ and can be deduced as:

$$
\begin{aligned}
\left(\frac{S}{N}\right)_{\nu} &= \sqrt{\frac{2}{K_{\mathrm{s}}}\frac{B(\nu)}{\overline{B}_{\mathrm{e}}}}\left(\frac{S}{N}\right)_{t=0} \\
&= \sqrt{\frac{2}{K_{\mathrm{s}}}\cdot\frac{\frac{1}{M}}{\frac{1}{K_{\mathrm{s}}}}}\cdot\frac{V}{\sqrt{1+V}}\cdot\sqrt{n} \\
&= \sqrt{2}\cdot\frac{1}{M}\cdot\frac{V}{\sqrt{1+V}}\cdot\sqrt{K_{\mathrm{s}}\cdot n} \\
&= \sqrt{2}\cdot\frac{1}{M}\cdot\frac{V}{\sqrt{1+V}}\cdot\sqrt{N_{\mathrm{CR}}\cdot T_{\mathrm{eff}}}
\end{aligned} \qquad (S.28)
$$

where M is the number of comb lines and K_{s} is the total scanned bins; N_{CR} is the counting rate. We denote T_{total} as total accumulation time, K as the number of bins for one frame of interferogram and L as the number of fringes for the scanned interferogram. Thus, the effective accumulation time, denoted as T_{eff}, can be represented as $\frac{K_{\mathrm{s}}}{K}\cdot T_{total}$ or $\frac{K_{\mathrm{s}}}{LK}\cdot T_{\mathrm{total}}$ when $K_{\mathrm{s}} < K$ or $K_{\mathrm{s}} > K$, respectively. Table S1 below shows the calculated theoretical SNR compared to the experimental valune.

Note 4. Comparison of different photon-counting configurations and the influence of fiber-length wandering

Fig. S8a depicts the configuration and protocol proposed in the main text. Here, we discuss the photon-counting DCS in two other possible configurations, which are shown in Fig. S8b and Fig. S8c, respectively. However, these two configurations lack the practical sensing ability or stability of coherent photon-counting statistics.

Fig. S8b shows a phase-sensitive configuration, where only the signal comb passes through the sensing path before it is combined with the local comb. The combined two combs are then separated into two channels by a beamsplitter. One channel is attenuated to photon level and detected by an SPD; the other channel is detected by a normal photodetector for the start signal production. According to Eq. (3) and Eq. (4) that $\varDelta r$, r_{a} and $\varOmega$ are also consistent across $\tilde{V}_{\mathrm{c}}(t)$ and $\tilde{V}_{\mathrm{d}}(t)$ in this configuration, it can maintain the long-term stability of coherent photon-counting accumulation. The experimental result shown in Fig. S9a verifies this photon-counting stability, and both the absorbance and dispersion spectra can be obtained in this asymmetric configuration (Fig. S9b). However, this configuration is impractical for practical applications. To produce the start signal, the power of the combined comb needs to be strong enough to be detected in the classical

frame. The detected photon-level signal is attenuated to what could have been captured using classical DCS. Therefore, this setup only serves as a demonstration experiment.

Fig. S8c shows another phase-sensitive configuration that separates the start signal production channel from the photon-level detection channel. This setup is capable of photon-level sensing in theory, but it lacks the stability of photon-counting statistics. Owing to extra internal paths introduced in this configuration, the time-delayed and phase shift terms in $\tilde{V}_{\mathrm{c}}(t)$ and $\tilde{V}_{\mathrm{d}}(t)$ can not remain consistent. Therefore, the start signals produced in this scheme fail to stay sync with the single-photon dual-comb interference signal, hindering the accurate reconstruction of the interference pattern. As shown in Fig. S9c, the substantial time delay caused by fiber length variations makes it challenging to discern the interference pattern through the accumulation process.

In contrast, our proposed method in the main text shows long-term stability of photon-counting statistics and practical sensing capability, enabling open-path photon-counting DCS.

3. Supplementary figures

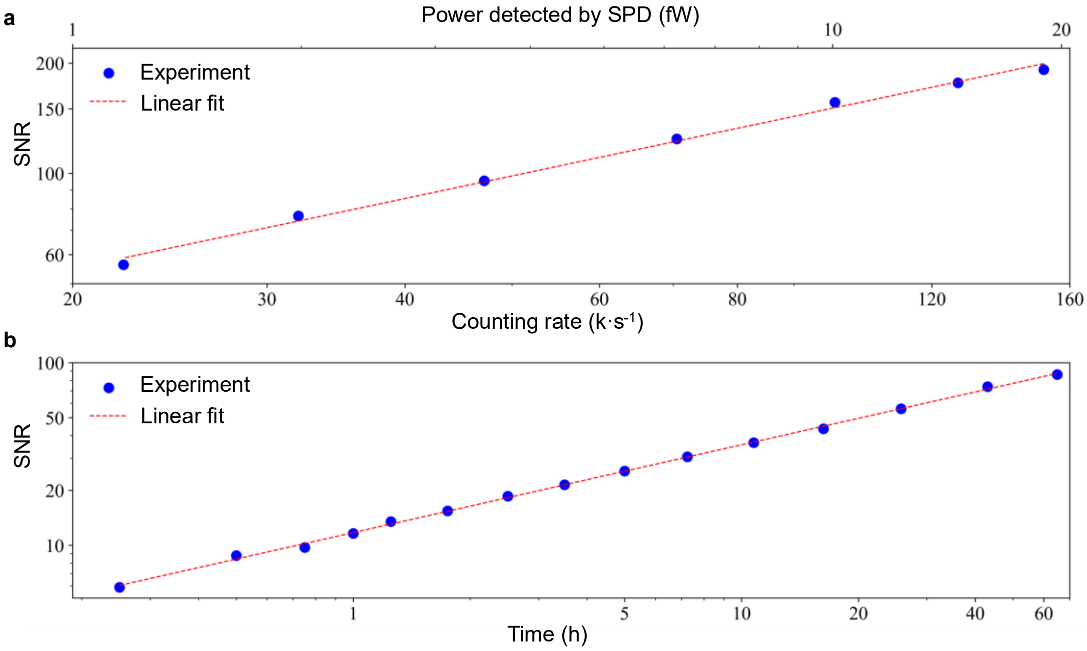

Fig. S1 Variation of the SNR of the photon-counting interferogram with photon counting rate and time. Both x- and y- axes are in log-scale. **a** The SNR is proportional to the square root of the photon counting rate. Each data point (blue circle) is acquired for an accumulation time of 4 hours. The fitting slope (red dashed line) is 0.68. We attributed this to the change in SPD's working conditions over the extended test period, such as dark counting rate and SPD bias voltage. **b** The SNR is proportional to the square root of the accumulation time over 65 hours, verifying the long-term stability of the photon-counting statistics. The fitting slope is 0.48. The counting rate of the SPD is fixed at 105.2 $k \cdot s^{-1}$, equivalent to 13.5 fW received by the SPD.

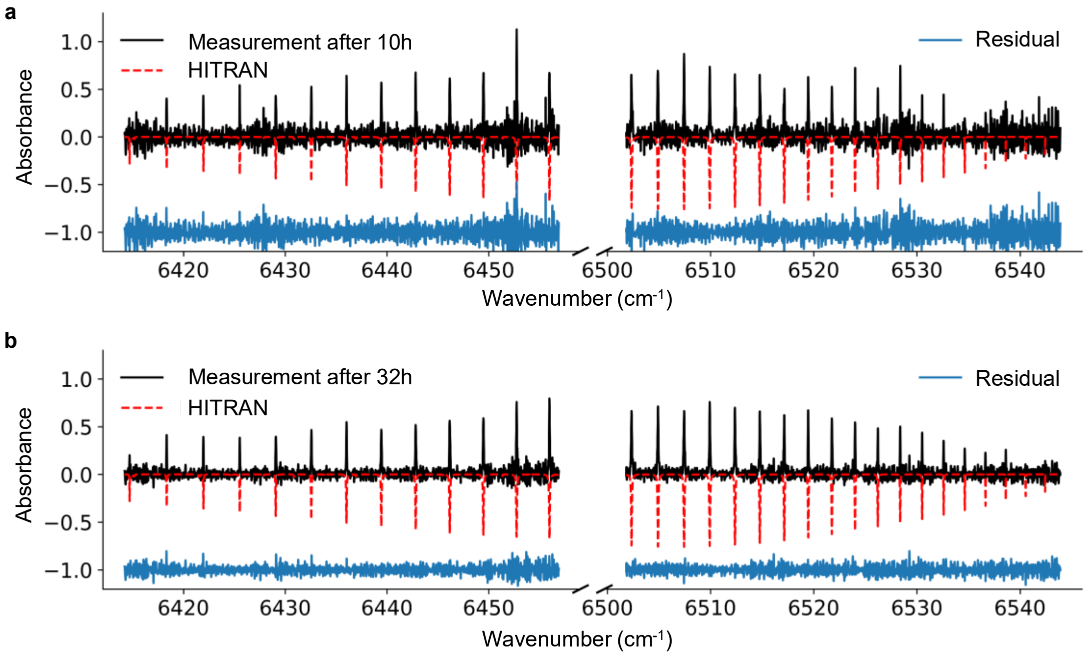

Fig. S2 Broadband (20nm) absorption spectra of $H^{13}C^{14}N$ after the accumulation time of 10 hours (a) and 32 hours (b), respecivelty. The spectra of $H^{13}C^{14}N$ were obtained with an average detection power of 4 attowatts per comb line (black solid lines). The theoretical spectra based on HITRAN2020 are also presented for comparison (red dashed line, inverted for clarity). The residual between the measured and calculated spectra are presented in blue lines, shifted by -1 for clarity.

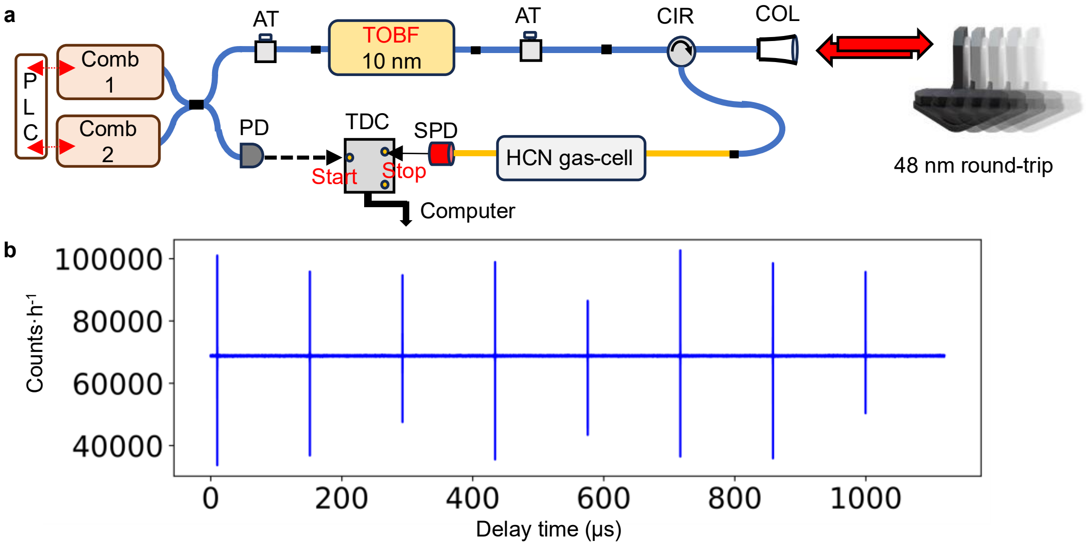

Fig. S3 a Experimental setup of broadband photon-counting DCS at an average detection power below 130 attowatts per comb line. The position of the HCN gas cell and 10 nm tunable optical bandwidth filter is switched to eliminate the birefringence interference effect. **b** The results of photon-counting statistics with 1200 μs scanning time. The photon-counting interferogram shows 8 clearly center fringes, indicating the comb-line-resolved spectrum.

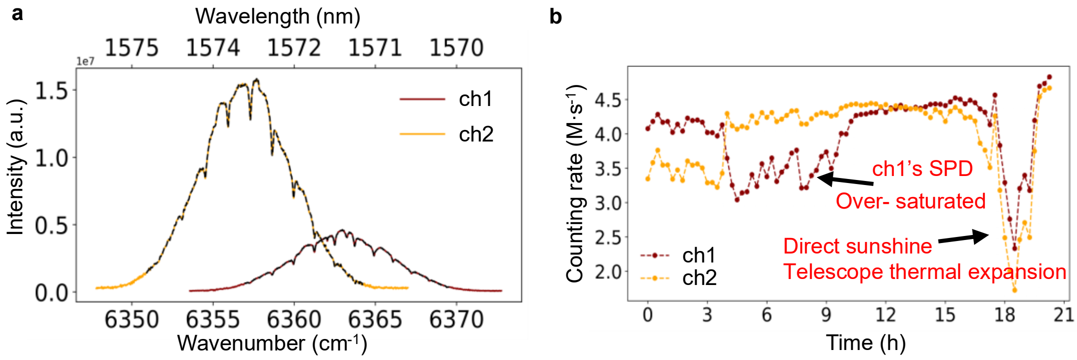

Fig. S4 Atmospheric spectra and working conditions of the SPDs in open-path photon-counting DCS experiments. a Transmittance spectra from both channels accumulated over 3 h from 10: 05 to 13:05 on 09/07. The y-axis is linear, and the dashed black lines represent spectral ranges used for absorbance extraction in Fig. 5a. **b** Real-time counting rate of the SPDs in both channels. Between 4 – 9.5 h (blue dashed boxes), the SPD in ch1 experienced saturation due to unexpectedly high atmospheric transparent condition. We adjusted the attenuation of ch1 at 9.5 h. Between 17.5 – 19.5 h in the afternoon (grey dashed box), direct sunlight caused thermal expansion of the telescope, temporarily reducing received power.

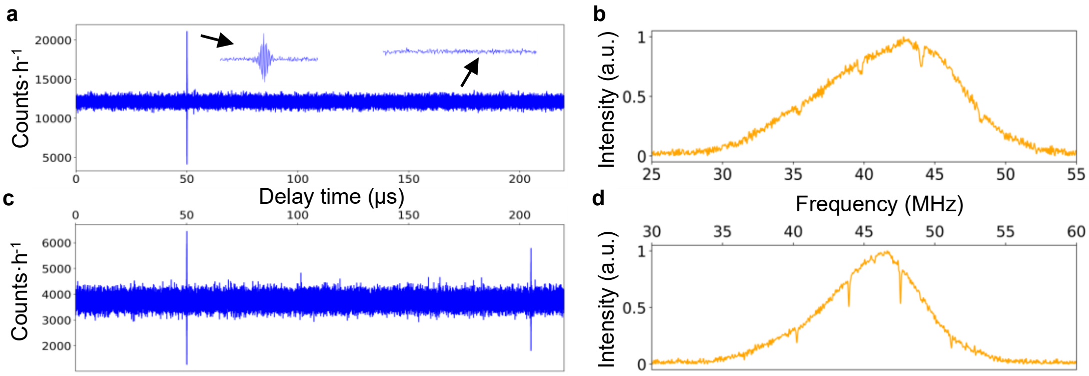

Fig. S5 a Photon-counting interferogram with the dual-comb source in a direct RF reference scheme; and **b** is the corresponding RF spectrum. The interferogram has a period of 119.8 μs. The second central fringe, expected around 170 μs in the delay time axis, is absent. **c** Photon-counting interferogram with dual-comb source in an optical reference scheme; and **d** is the corresponding RF spectrum. The two central fringes are depicted. The HCN absorption features in **b** are pronounced and narrower compared to that in **d**, due to the difference in spectral resolution.

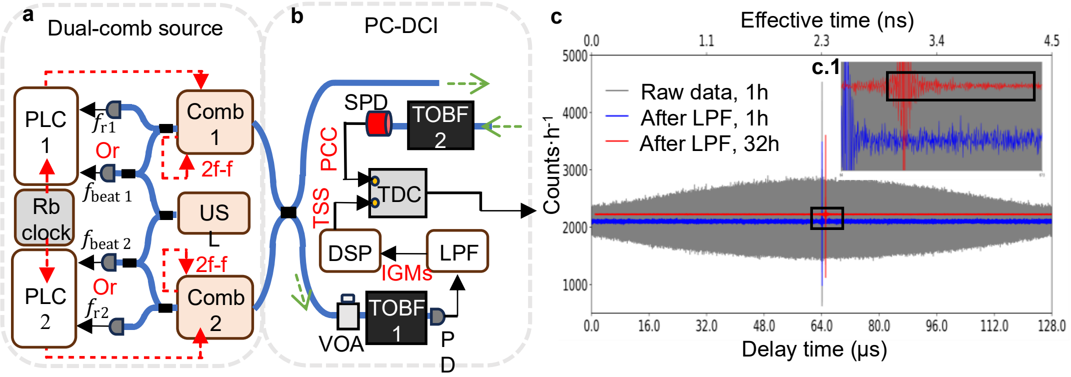

Fig. S6 Experimental setup of the dual-comb source (a) and the photon-counting dual-comb interferometry (b). The results of photon-counting statistics are shown in c. The grey line in **c** represents the original raw data after 1 hour of accumulation time. The interferogram (red line) is obtained after applying a digital low-pass filter to the raw data, shifted by 1 and 150 counts for clarity. The interferogram after 32 hours of accumulation time shows a better SNR (blue line). **c.1** shows the magnified view of **c**. **USL,** ultra-stable laser; **PLC**, phase-lock control unit**;** **SYN**, clock synchronization; **SPD**, single-photon detector; **PCC**, photon-counting clicks; **TDC**, time-to-digital convert; **TOBF**, tunable optical bandpass filter; **VOA**, variable optical attenuator; **PD,** photodetector**;** **LPF**, low-band pass filter; **DSP**, digital signal processor; **IGMs**, interferograms; **TSS**, timestamp of the start signal; **CIR**, circulator; **COL**, collimator.

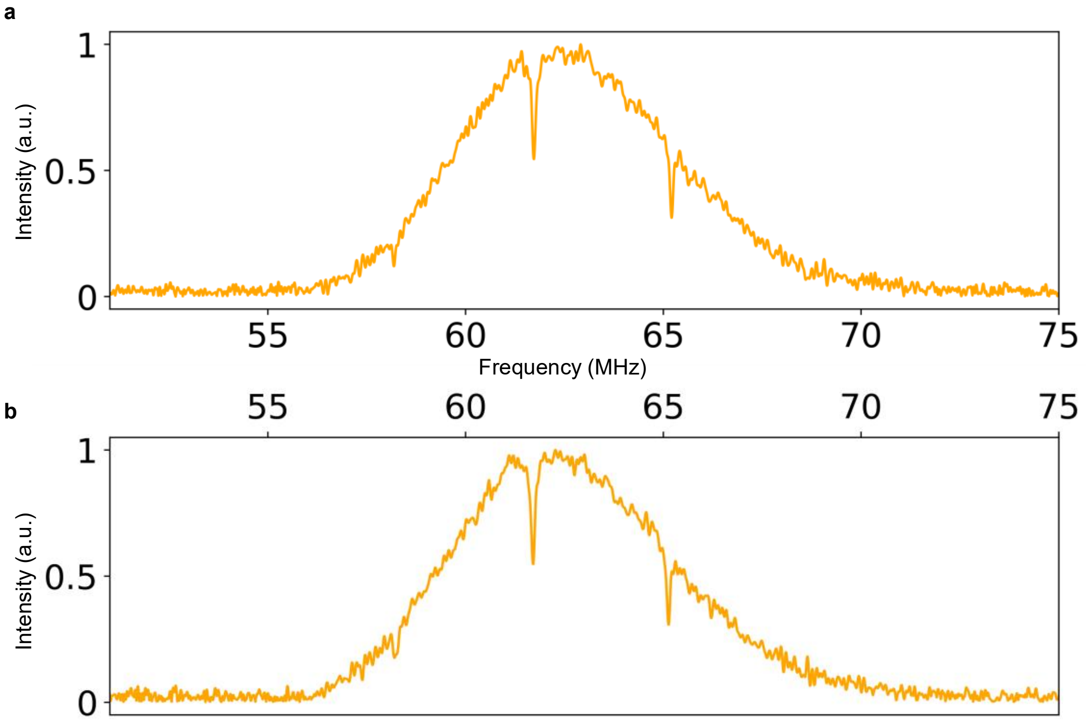

Fig. S7 Photon-counting RF spectra measured under different non-zero values of $\boldsymbol{\Delta\varphi_{ceo}}$. Given that $f_r/\Delta f_r$ is not an integer in our comb system, different non-zero values of $\Delta\varphi_{ceo}$ are realized by adjusting the polarity for the carrier-envelop frequency (f_{ceo}) of the two OFCs, i.e., **(a)** $f_{ceo,1} = f_{ceo,2}$, and **(b)** $f_{ceo,1} = -f_{ceo,2}$.

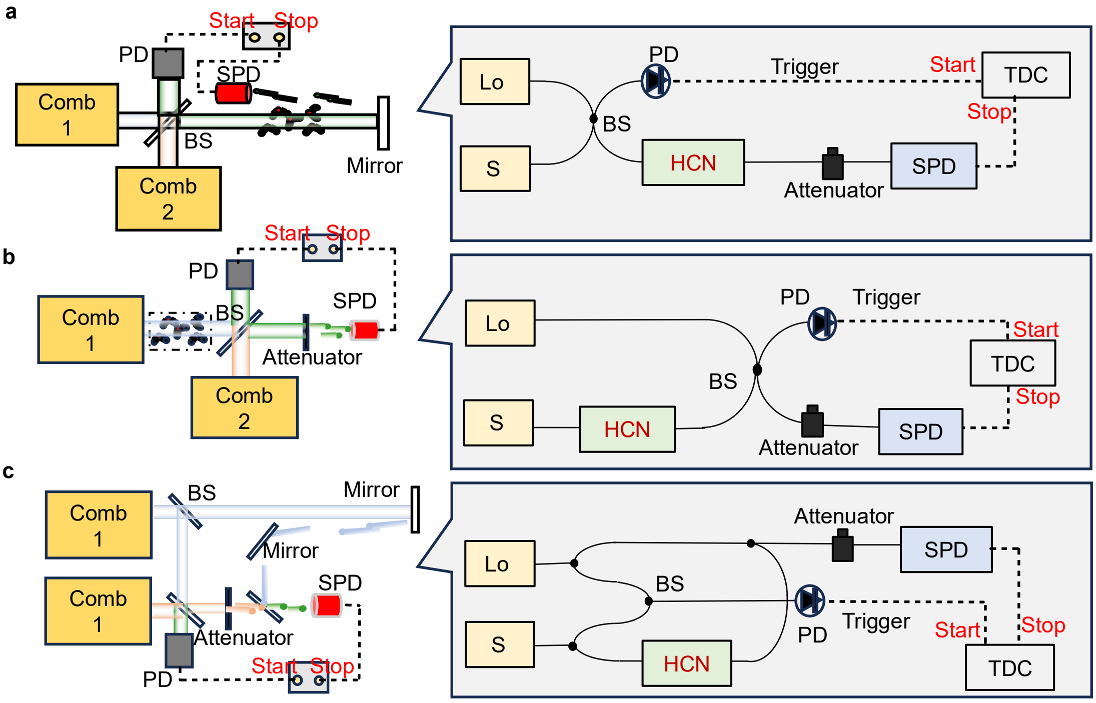

Fig. S8 Configurations of three types of photon-counting dual-comb interferometry. The experimental setups of the developed all-fiber system based on these three configurations are provided on the right side.

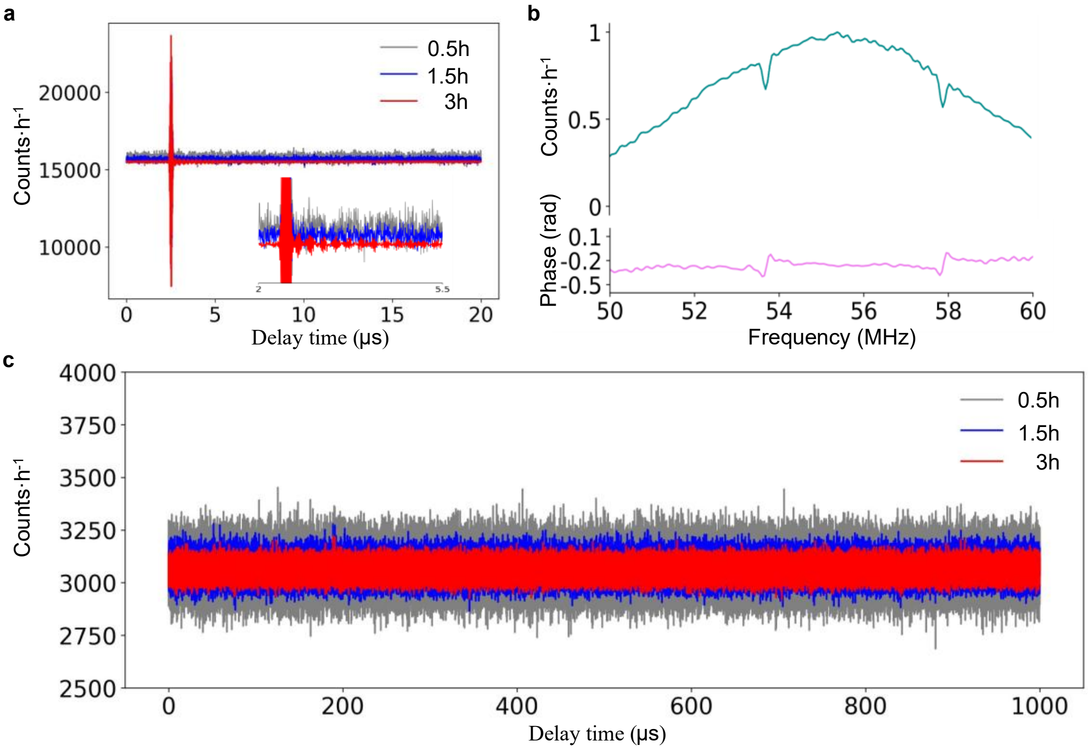

Fig. S9 Photon-counting statistics obtained using the detection configurations depicted in Fig. S8b (a and b) and Fig.S8c (c).

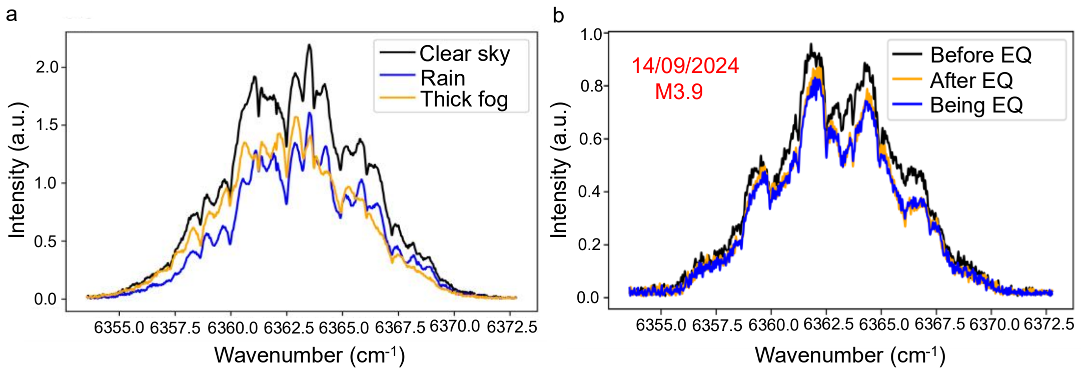

Fig. S10 Open-path photon-counting DCS conducted under extreme conditions (rain, fog, and seismic events). Under these conditions, the laboratory windows were closed for safety consideration. Hence, the measurement includes the absorption spectrum of the window glass. **a** The transmittance spectra obtained under different weather conditions. The black, blue, and orange solid lines represent the measurement conditions with clear sky (3:00 – 6:00, UT+8 hereafter), heavy rain (9:00 – 12:00), and thick fog (20:00 -23:00,) on 09/11/ 2024. The y-axis is in a linear scale. **b** The 15-min accumulated transmittance spectra before (black lines), during (blue lines) and after (orange lines) a magnitude-3.9 earthquake (EQ) on 5:12, 14/09/2024.

Table S1. Comparison of the measured and theoretical shot-noise-limit SNR. Discrepancies between the theoretical and experimental SNR values can be attributed to the additive noise present in the experimental setup and variations in the intensity of comb lines, which are not taken into account in the theoretical analysis. The average power per comb line is determined by halving the total detected power (derived from the photon counting rate and the SPD's quantum efficiency of 35.5%) and then dividing this value by the number of comb lines.

		M	V	Scan time T_{scan} (μs)	L	T_{total} (h)	Count rate N_{cR} (M·s^{-1})	power detect. by SPD (fW)	Aver. Power per comb line before SPD (aW)	Theor. SNR	Exp. SNR
**Fig. 3a**	P	6413	0.67	128	1	10	0.148	19.2	4.2	8.3	7.4
	R	6427	0.61				0.144	18.7	4.1	7.5	6.2
**Fig. 3c**		2154	0.69	7500	48	7	0.162	20.8	22.2	17	14
**Fig. 4a**	P	6413	0.51	1120	8	10	4.28	547.8	120	36	33
	R	6427	0.64				4.48	580.0	127	43	31
**Fig. 5a (ch)**	1	2827	0.78	1120	8	3	4.42	557.6	278	66	55
	2	2254	0.83		8		4.33	558.3	342	83	81
